# Biometabolites of *Citrus unshiu* Peel Enhance Intestinal Permeability and Alter Gut Commensal Bacteria

**DOI:** 10.3390/nu15020319

**Published:** 2023-01-09

**Authors:** Se-Hui Lee, Dongju Seo, Kang-Hee Lee, So-Jung Park, Sun Park, Hyeyun Kim, Taekyung Kim, In Hwan Joo, Jong-Min Park, Yun-Hwan Kang, Gah-Hyun Lim, Dong Hee Kim, Jin-Young Yang

**Affiliations:** 1 Department of Integrated Biological Science, Pusan National University, Busan 46241, Republic of Korea; 2 Department of Biology Education, Pusan National University, Busan 46241, Republic of Korea; 3 Department of Pathology, College of Korean Medicine, Daejeon University, Daejeon 34520, Republic of Korea; 4 Department of Industry Promotion, National Institute for Korean Medicine Development, Geongsan 38540, Republic of Korea; 5 Department of Biological Sciences, Pusan National University, Busan 46241, Republic of Korea

**Keywords:** natural extract, *Citrus unshiu* peel, dextran sodium sulfate, inflammatory bowel disease, intestinal tight junction, T helper 17 cell, commensal microbiota

## Abstract

Flavanones in *Citrus unshiu* peel (CUP) have been used as therapeutic agents to reduce intestinal inflammation; however, the anti-inflammatory effects of their biometabolites remain ambiguous. Here, we identified aglycone-type flavanones, such as hesperetin and naringenin, which were more abundant in the bioconversion of the CUP than in the ethanol extracts of the CUP. We found that the bioconversion of the CUP induced the canonical nuclear factor-κB pathway via degradation of IκB in Caco-2 cells. To check the immune suppressive capacity of the aglycones of the CUP in vivo, we orally administered the bioconversion of the CUP (500 mg/kg) to mice for two weeks prior to the 3% dextran sulfate sodium treatment. The CUP-pretreated group showed improved body weight loss, colon length shortage, and intestinal inflammation than the control mice. We also found a significant decrease in the population of lamina propria Th17 cells in the CUP-pretreated group following dextran sodium sulfate (DSS) treatment and an increase in mRNA levels of occludin in CUP-treated Caco-2 cells. Pyrosequencing analysis revealed a decreased abundance of *Alistipes putredinis* and an increased abundance of *Muribaculum intestinale* in the feces of the CUP-pretreated mice compared to those of the control mice. Overall, these findings suggest that the pre-administration of CUP biometabolites may inhibit the development of murine colitis by modulating intestinal permeability and the gut microbiome.

## 1. Introduction

In recent years, natural extracts have been used as therapeutic agents worldwide [1,2,3,4,5]. Most of them have antimicrobial activities against external pathogens [6,7] and are being used to treat various diseases, such as acute gastroenteritis [8], diabetes mellitus [9], and obesity [10] in clinical trials. Although raw natural products are beneficial, the derivatives of pharmaceutically active components are more important for further clinical application. Natural product derivatives accounted for the largest portion of approved antibacterial drugs until 2019 worldwide [11]. For this reason, many advanced techniques, such as microwave-assisted extraction (MAE), ultrasound-assisted extraction (UAE), and pressurized liquid extraction methods, have been developed owing to the increased interest in natural products [12]. There are numerous extraction solvents for obtaining chemical compounds, such as methanol, ethanol, and acetone extracts [12], from natural sources, suggesting that the final compounds obtained via different extraction methods may have various therapeutic applications.

As a valuable natural source, *Citrus (C.) unshiu* has been studied in diverse clinical fields. In particular, the therapeutic effects of the peel covering *C. unshiu* have received more attention than other parts of the plant, because it exerts outstanding antioxidant effects compared to the pulp owing to its high mineral composition [13,14]. *C. unshiu* peel is widely used as a therapeutic agent for clinical purposes, such as neuropathic pain [15], cervical cancer [16], nonalcoholic fatty liver [17], hyperglycemia [18], pancreatic cancer [19], and systemic inflammation [20]. This raw material can also be processed using various extraction protocols, as mentioned above. Especially, water or ethanol extraction is a commonly used method to extract flavonoids from natural substances, hence, many studies employing solvent-extracted CUP have been conducted. For example, the ethanol derivative of CUP ameliorated oxidative stress by suppressing nicotinamide adenine dinucleotide phosphate (NADPH) oxidase 2 expression (NOX2) in a chemotherapy-induced neuropathic pain animal model [15] and decreasing the expression levels of pro-inflammatory cytokines, such as interleukin (IL)-6, interferon (IFN)-γ, and tumor necrosis factor-alpha (TNFα), in a type 2 diabetes and systemic inflammation mouse model [18,20]. Moreover, water extracts of CUP have been reported to attenuate dextran sulfate sodium-induced colitis by regulating the phosphoinositide 3-kinase (PI3K)/Akt signaling pathway [21]. This prompted us to explore other pharmaceutically active products in CUP. However, little is known regarding the therapeutic effects of CUP extracted via bioconversion on intestinal homeostasis.

Citrus flavonoids, such as hesperidin and narirutin, are converted to their aglycones by metabolites secreted from the gut microbiota, followed by absorption through the intestinal epithelium [22,23]. Notably, intestinal epithelial cells (IECs) play several pivotal roles in recognizing gut microbes and blocking external stimuli, such as antigens and microorganisms [24]. Numerous receptors expressed on the intestinal epithelium, such as Toll-like receptors (TLRs) and NOD-like receptors (NLRs), interact with microbial signals in a bidirectional manner, eliciting an innate immune response and remodeling the bacterial configuration [25,26,27]. In addition, tight junction complexes located on the apical side of IECs block the influx of components present in the luminal environment into the lamina propria across the intestinal epithelium [28]. Any disturbance in gut microbes following the loss of intestinal barrier function can provoke intestinal inflammation or induce an imbalance of T-cell subsets [29,30], suggesting that the maintenance of the gut microbiota composition and epithelial tight junctions by natural products is crucial for intestinal immune homeostasis.

Inflammatory bowel disease (IBD), including ulcerative colitis (UC) and Crohn’s disease (CD), is a representative bowel disorder characterized by an irregular inflammatory response in the gastrointestinal (GI) tract, eliciting symptoms such as weight loss, rectal bleeding, and diarrhea [31]. Although the specific cause of IBD has not been elucidated yet, the pathogenic mechanisms of IBD are strongly suggested to involve microbial imbalance and abnormal immune reactions in the GI tract [32]. In particular, numerous studies have reported that the abundance of beneficial bacteria, including *Firmicutes*, *Bifidobacterium*, *Clostridium* (Ⅳ and ⅨⅤ), *Lactobacillus*, and *Lachnospiracea*, is decreased and that of harmful bacteria, including *Enterobacteriaceae*, *Proteobacteria*, *Fusobacterium*, and *Bacteroides*, is increased in patients with CD and UC [33,34]. Furthermore, IECs are crucial for the formation of the intestinal barrier, implying that the disruption of the junction between IECs may induce gut inflammation. These factors are not independent of each other because compositional changes in the gut microflora can affect the IECs and regulate the differentiation of T helper 17 (Th17) cells [35,36]. Thus, many trials have been conducted to determine the therapeutic effects of natural products on IBD based on their anti-inflammatory and anti-oxidative activities [37,38,39,40,41]. For instance, total extracts of *Abelmoschus manihot* (L.) ameliorate DSS-induced colitis by decreasing the number of Th17 cells [42] and colonic inflammation-associated fibrosis by suppressing extracellular matrix accumulation [43]. In addition, phytochemicals derived from plants also mitigate colonic inflammation by balancing T-cell populations and promoting gut microbiota to produce butyrate [44], which has beneficial effects on IBD [45]. The antimicrobial effects of CUP against pathogenic bacteria are remarkable, as patients with active IBD are vulnerable to the entry of microorganisms through the loose intestinal barrier [46].

In this study, we examined the anti-inflammatory effects of CUP on Caco-2 epithelial cells and in a DSS-induced colitis murine model. We observed an improvement in intestinal inflammation and a decrease in colonic Th17 cells in the CUP-pretreated mice after 3% DSS treatment, accompanied by increased *Occludin* levels in Caco-2 and changes in bacterial composition. Our data suggest that CUP metabolites enhance intestinal permeability and alter the gut commensal bacteria to prevent colonic inflammation, thereby regulating intestinal homeostasis in Th17 cells.

## 2. Materials and Methods

### 2.1. Ethics Statement

All mice were housed under specific pathogen-free conditions in the experimental facility at Pusan National University (Busan, Korea) and provided sterilized diet and water ad libitum. All animal experiments were approved by the Institutional Animal Care and Use Committee of Pusan National University (approval no: PNU-2022-0123), and all efforts were made to minimize the suffering of the animals.

### 2.2. Mice and Natural Products

Six-week-old female C57BL/6 mice were purchased from Samtako Bio-Korea, Inc. (Osan, Korea). The CUP extracts were provided by the College of Korean Medicine (Daejeon, Korea). The CUP was diluted in 200 μL of phosphate-buffered saline (PBS) and administered to mice (500 mg/kg) via an oral gavage using intubation needles for two weeks. The control group was administered with the same volume of PBS as the control group.

### 2.3. Extraction of CUP

The CUP extract used in this experiment was obtained by adding 10 times the volume of 70% ethanol to 100 g at room temperature for 24 h. The supernatant was filtered using a microfilter with a 1 µm pore size, the remaining residue was extracted two more times under the same conditions, and a total 54 g of a dried product (yield 54%) was obtained via concentration (Rotary Evaporator NVC-2100; EYELA, Tokyo, Japan) and drying (Labconco Freeze Drier.5; Labconco Corp., Kansas City, MO, USA). Ten milliliters of distilled water was added to 10 g of bran, placed in a 500 mL flask, mixed well, sterilized at 121 °C for 30 min, inoculated with Aspergillus kawachii, and cultured at 30 °C for three days. Fermented bran was suspended in 100 mL of 100 mM sodium phosphate buffer (pH 7.0), left at 4 °C for 18 h, filtered with a gauze, and the filtrate was centrifuged at 10,000 rpm for 15 min. The supernatant was used as the crude enzyme solution. After setting the optimal reaction conditions (30 °C, 24 h) using Aspergillus crude enzyme solution optimized for carbohydrase production conditions, 70% ethanol extract of CUP was reacted, and bioconversion metabolites were obtained.

### 2.4. Component Extraction from Natural Products

The compositional analysis was conducted using a Waters e2695 HPLC System/2998 PDA detector (Waters Corporation, Milford, MA, USA) equipped with a Waters Xbridge C18 (4.6 × 250 mm, 5 µm) column.

### 2.5. Antibodies and Reagents

The following antibodies were used for Western blotting: α-tubulin (catalog no. 2144S; Cell Signaling Technology, Danvers, MA, USA), p65 (clone D14E12, catalog no. 8242S; Cell Signaling Technology), phospho-p65 (Ser536) (clone 93H1, catalog no. 3033S; Cell Signaling Technology), IκBa (clone 44D4, catalog no. 4812S; Cell Signaling Technology), phospho-IκBa (Ser32) (clone 14D4, catalog no. 2859S; Cell Signaling Technology), and p105/p50 (clone D7H5M, catalog no. 12540S; Cell Signaling Technology). The antibodies used for the flow cytometry were CD3 (clone 17A2, catalog no. 100222; BioLegend, San Diego, CA, USA), CD4 (clone GK1.5, catalog no. 48-0041-82; eBioscience, San Diego, CA, USA), CD25 (clone PC61.5, catalog no. 12-0251-81; eBioscience), IFN-g (clone XMG1.2, catalog no. 12-7311-81; eBioscience), IL-17a (clone eBio17B7, catalog no. 17-7177-81; eBioscience), and Foxp3 (clone FJK-16S, catalog no. 17-5773-80; eBioscience).

### 2.6. DSS-Induced Colitis

The mice were administered 3% DSS (MP Biomedicals, CA) ad libitum in drinking water for eight days, which was changed to tap water on the last day. The food uptake and DSS intake per mouse were recorded. The body weight was monitored daily.

The disease activity score (DAI) was calculated as previously described [47]. Briefly, DAI was measured during the DSS treatment by monitoring the body weight loss, stool consistency, and gross bleeding. The parameters used for DAI were as follows: weight loss (0: none, 1: 1–5%, 2: 5–10%, 3: 10–20%, and 4: >20%), stool consistency (0: normal, 1: loose, and 4: diarrhea), and gross bleeding (0: absence, 2: blood-tinged, and 4: presence). The formula used for the DAI calculation was as follows:

DAI = (weight loss + stool consistency + gross bleeding)(1)

### 2.7. Histological Analysis

The large intestine was obtained from mice, and all mesenteric fat was removed. Then, the whole intestine was cut longitudinally and unfolded into a rectangle to roll the intestine into a Swiss roll. The tissues rolled from the distal to proximal part of the intestine were fixed in 4% formalin at 4 ℃ overnight. The fixed tissues were consecutively dehydrated in 70, 80, 90, 95%, and 100% ethanol and in xylene and embedded in paraffin. The paraffin-embedded samples were cut into 6 μm sections using a microtome (Leica Biosystems, Wetzlar, Germany), attached on glass slides for staining with hematoxylin (YD Diagnostics, Gyeonggi-do, Korea) and eosin (Wako, Osaka, Japan) and periodic acid-Schiff staining (Sigma-Aldrich, St. Louis, MO, USA), and observed under a digital inverted light microscope (EVOS, Thermo Fisher Scientific, Waltham, MA, USA). The histopathological scores were measured as described previously [47]. Briefly, each specimen was judged based on the severity of inflammation (0: none, 1: slight, 2: moderate, and 3: severe), extent of injury (0: none, 1: mucosal, 2: mucosal and submucosal, and 3: transmucosal), and crypt damage (0: none, 1: basal one-third damaged, 2: basal two-thirds damaged, 3: only surface epithelium intact, and 4: entire crypt and epithelium lost), and the average was calculated.

### 2.8. cDNA Synthesis and Real-Time PCR

The total RNA was extracted from Caco-2 cells using TRIzol reagent (Sigma-Aldrich), and 1 μg of mRNA was converted into cDNA with superscript II reverse transcriptase (Invitrogen, Carlsbad, CA, USA). The real-time PCR was performed to determine the relative mRNA expression levels using the CFX Connect Real-Time PCR Detection System (Bio-Rad, Hercules, CA, USA) and PowerUP SYBR Green Master Mix (Thermo Fisher Scientific). All reactions were performed in the same manner: 95 °C for 3 min, followed by 45 cycles of 95 °C for 10 s and 60 °C for 30 s. The mRNA expression levels were normalized to those of β-actin as a reference gene. All specific primer sets are listed in Table S2.

### 2.9. Cell Isolation and Analysis

The full length of the large intestine was removed from mouse adipose tissues, and all contents were eliminated. The tissues were cut into 1 cm sections and reacted with 1 mM EDTA RPMI medium (2% fetal bovine serum (FBS)) for 30 min at 37 °C on shaker at 280 rpm. Then, the tissues were washed with prewarmed PBS four times vigorously and chopped into 2–3 mm pieces. Subsequently, the tissues were processed in RPMI (10% FBS, collagenase, and DNase) for 30 min at 37 °C on shaker at 800 rpm. The supernatant was centrifuged at 1200 rpm and 4 °C for 5 min after filtering using a cell strainer, and this process was performed twice. The cell pellet was suspended in 4 mL of 40% Percoll, loaded on 2 mL of 75% Percoll using a disposable spoid, and centrifuged at 2000 rpm at room temperature with no brake. After the removal of debris, the cells were harvested in a 15 mL tube filled with RPMI (2% FBS) and centrifuged at 1200 rpm and 4 °C for 5 min to obtain cells. For intracellular staining, the T cells isolated from the large intestine were stimulated for 5 h with phorbol myristate acetate (50 ng ml^−1^) plus ionomycin (0.5 ug ml^−1^) in the presence of monensin (eBioscience).

### 2.10. Mucin2 (MUC2) Immunofluorescence Staining

For Carnoy’s fixation, 1 cm of colon was dissected from the mouse and unfolded into a square shape. The tissues were fixed using Carnoy’s fixative solution (10% glacial acetic acid, 30% chloroform, 60% ethanol, and 1 g ferric chloride) and embedded in frozen section medium (Leica Biosystems, Nussloch, Germany) at −80 °C for 1 h before storage at –20 °C. For further staining, we sectioned blocks into 6 μm using a cryotome (Leica Biosystems), fixed them in precooled (−20 °C) acetone for 2 min, and washed them in fresh PBS for 5 min. The tissues were incubated with Fc blocking antibody (BD Biosciences, San Jose, CA, USA) for 30 min at room temperature and then with anti-Muc2 antibody (Abcam, Cambridge, UK) for 2 h at room temperature as well. After rinsing in fresh PBS, the tissues were incubated with Alexa Fluor 488-conjugated anti-IgG antibody (Abcam), counterstained with NucRed Live 647 ReadyProbes Reagent (Invitrogen, Carlsbad, CA, USA), and observed under a Leica DMi8 microscope (Leica Microsystems, Wetzlar, Germany).

### 2.11. Immunoblotting

The cells were lysed with the radioimmunoprecipitation assay (RIPA) buffer (50 mM Tris-HCL (pH 7.4), 150 mM NaCl, 1% (*v*/*v*) NP-40, 0.5% (*w*/*v*) Na-deoxycholate, 1 mM EDTA (pH 7.4)), containing 5 mM NaF, 1 mM PMSF, and 1 mM DTT. The protein lysates were solubilized in 4× protein digestion buffer (0.1 M Tris-HCl (pH 6.8), 4% (*w*/*v*) sodium dodecyl sulfate (SDS), 20% (*v*/*v*) glycerol, 0.001% (*w*/*v*) bromophenol blue, and 200 mM β-mercaptoethanol). Whole-cell protein extracts were separated by SDS-polyacrylamide gel electrophoresis (10% gel) and transferred to a polyvinylidene fluoride membrane (0.45 µm; Millipore, Burlington, MA, USA). After blocking for 1 h with 5% skim milk (BD, Franklin Lakes, NJ, USA) containing 0.05% Tween-20 in TBS, the membranes were washed thrice with 0.05% Tween-20 in TBS and probed with primary antibodies overnight at 4 °C. The membranes were then probed with goat anti-rabbit (Jackson, 1:10,000) IgG secondary antibodies conjugated with horseradish peroxidase for 1 h. Bands were visualized using chemiluminescence detection (ECL, Millipore, MA, USA) and normalized by α-tubulin.

### 2.12. Microbiome Analysis

The stool samples were collected from individual mice freshly on the 14th day after treatment with CUP or PBS. The gDNA was isolated from the stool samples using QIAamp Fast DNA Stool Mini Kits (Qiagen, Valencia, CA, USA) and amplified via PCR using primers targeting the V3 to V4 region of 16S rRNA (forward: 5’-TCGTCGGCAGCGTCAGATGTGTATAAGAGACAGCCTACGGGNGGCWGCAG-3’, reverse: 5’-GTCTCGTGGGCTCGGAGATGTGTATAAGAGACAGGACTACHVGGGTATCTAATCC-3’). The cycling protocol for the 1st PCR was 95 °C for 3 min, followed by 25–30 cycles of 95 °C for 30 s, 55 °C for 30 s, 72 °C for 30 s, and 72 °C for 5 min. The 1st PCR product was purified using AMPure beads (Agencourt Bioscience, Beverly, MA, USA). Following purification, 2 µL of the 1st PCR product was again PCR amplified for the final library construction containing the index using NexteraXT Indexed Primer. The cycle conditions for the 2nd PCR were the same as those for the 1st PCR, except for 10 cycles. The PCR products were purified using AMPure beads. The final purified product was quantified using qPCR according to the qPCR Quantification Protocol Guide (KAPA Library Quantification kits for Illumina Sequencing platforms) and qualified using the TapeStation D1000 Screen Tape (Agilent Technologies, Waldbronn, Germany). Paired-end (2 × 300 bp) sequencing was performed by Macrogen using the MiSeq™ platform (Illumina, San Diego, CA, USA).

### 2.13. Statistical Analysis

All data are represented as the mean ± standard error of the mean (SEM) and analyzed statistically using GraphPad Prism software 7.0 (GraphPad, San Diego, CA, USA). Body weight change and DAI were analyzed using two-way analysis of variance (ANOVA). Significant differences between two groups were determined using an unpaired one- or two-tailed *t*-test (* *p* < 0.05, ** *p* < 0.01, and *** *p* < 0.001; ns, not significant).

## 3. Results

### 3.1. Bioconversion Metabolites in CUP Mainly Consist of Hesperetin and Naringenin

**Figure 1:**
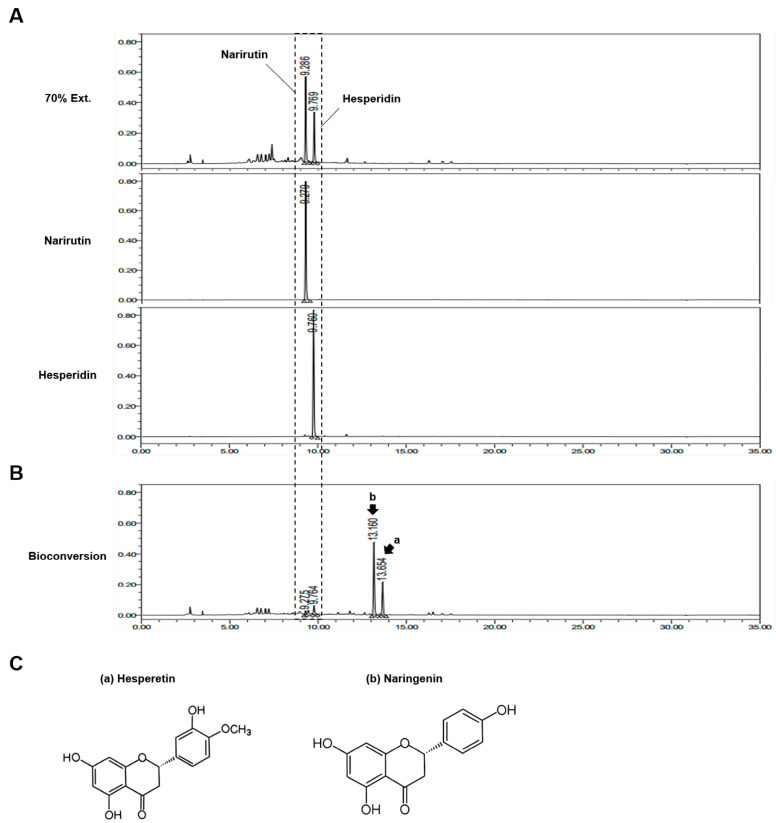
Compound analysis of Citrus unshiu peel (CUP). To identify the components of CUP, two kinds of metabolites extracted from the CUP using 70% ethanol (**A**) and bioconversion (**B**) were analyzed via high-performance liquid chromatography (HPLC). (**C**) Chemical structures of hesperetin and naringenin.

To determine the flavonoid present in CUP, we analyzed the chemical components using high-performance liquid chromatography (HPLC) and confirmed that the solvent extracts of the CUP included narirutin and hesperidin (Figure 1A), which are flavonoids abundant in citrus fruits. Interestingly, when we examined the composition of the bioconversion of the CUP, two different peaks from those of narirutin and hesperidin were observed (Figure 1B). These peaks were found to correspond to naringenin and hesperetin, which are aglycones (Figure 1C), a type of residue after the deconjugation of the sugar group from the glycoside of narirutin and hesperidin, respectively. Therefore, these data suggest that the bioconversion metabolites of the CUP have a distinct mechanism from that of ethanol extracts of the CUP due to the fact of their different chemical properties.

### 3.2. Bioconversion Metabolites in CUP Can Reduce Intestinal Inflammation Mediated by IκB Degradation

**Figure 2:**
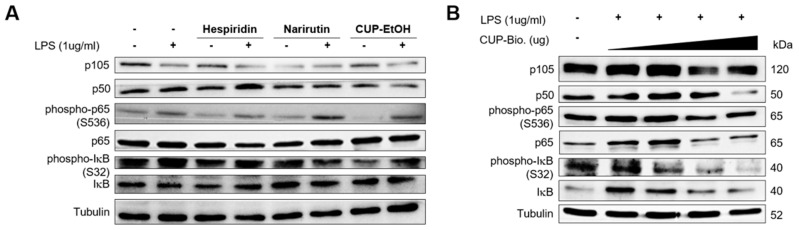
Bioconversion of Citrus unshiu peel (CUP) suppresses nuclear factor (NF)-κB activation in Caco-2 cells. Immunoblotting analysis of canonical NF-κB proteins in whole-cell lysates of Caco-2 cells. Caco-2 cells were treated with hesperidin, narirutin (50 µg/mL), CUP extracted by ethanol (1 mg/mL) (**A**) and bioconversion of CUP (0, 100, 500, and 1000 mg/mL) (**B**) for 1 h, followed by stimulation with LPS (1 µg/mL) for 2 h. After stimulation, cells were harvested for immunoblotting.

We examined the effects of the bioconversion and ethanol extract of the CUP on NF-κB activation in intestinal epithelial Caco-2 cells via immunoblotting. We found that the ethanol extract of CUP moderately reduced the expression levels of p50 and phosphorylation of IκB induced by lipopolysaccharide (LPS), whereas hesperidin and narirutin, which are flavonoid components of the ethanol extract of the CUP, also decreased the phosphorylation of IκB but not the expression of p50 (Figure 2A). Interestingly, the bioconversion of the CUP drastically repressed the expression of p50 and the phosphorylation of IκB in a dose-dependent manner (Figure 2B). Taken together, CUP blocked canonical NF-κB activation by inhibiting the expression of p50 and phosphorylation of IκB in Caco-2 cell. These anti-NF-κB effects were more dramatic in the bioconversion of the CUP than in the ethanol extract.

### 3.3. Bioconversion of CUP Ameliorates the Inflammatory Phenotypes in the DSS-Induced Colitis Model

**Figure 3:**
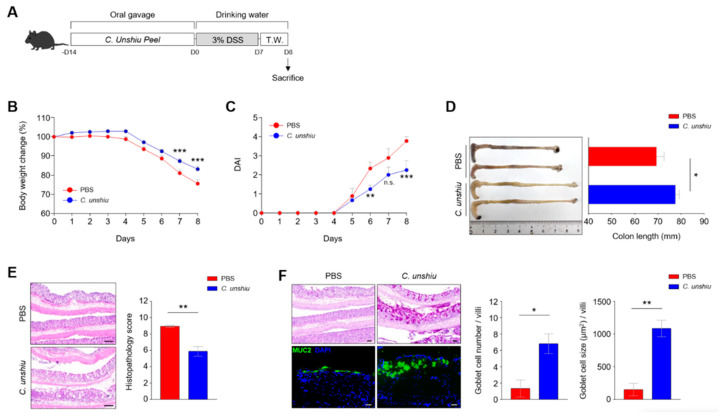
Citrus unshiu peel (CUP) ameliorates dextran sulfate sodium (DSS)-induced inflammation in vivo. (**A**) Scheme of the treatment with CUP and 3% DSS water in murine model. Body weight change (**B**) and fecal pathogenicity was monitored, and the disease activity index (DAI) (**C**) was evaluated for eight days during the induction of colitis. Representative image of the colon length (**D**; **left**) and its quantification bar graph (**D**) (**right**) with phosphate-buffered saline (PBS) and CUP-treated mice. The large intestine of the PBS- and CUP-treated mice were analyzed via hematoxylin and eosin (H&E) staining (**E**), periodic acid-Schiff (PAS) staining, and immunofluorescence (IF) staining for mucin 2 (MUC2) (**F**) and shown as representative images and quantification bar graphs. All data are represented as the mean ± standard error of the mean (SEM) of independent individuals. Statistical analyses were conducted using two-way analysis of variance (ANOVA) with Sidak’s multiple comparisons test for body weight change and DAI and the unpaired *t*-test for colon length and histology score. * *p* < 0.05, ** *p* < 0.01, and *** *p* < 0.001; n.s., not significant. Scale bar = 100 μm.

To investigate the protective effects of the bioconversion of the CUP on colitis, seven-week-old C57BL/6 mice were administered with the bioconversion of the CUP (500 mg/kg) for two weeks and then treated with 3% DSS water for 1 week (Figure 3A). Interestingly, mice pretreated with CUP were protected against severe body weight loss compared to those in the PBS-treated group (Figure 3B). In addition, the CUP-treated group showed moderate disease activity scores and recovered colon length following 3% DSS treatment (Figure 3C,D). We confirmed the anti-inflammatory effects of the CUP using several histological analyses in terms of partially restored epithelium and increased mucin production in the CUP-treated mice (Figure 3E,F). These findings suggest that CUP biometabolites can improve intestinal inflammation symptoms in mice with DSS-induced colitis.

### 3.4. Bioconversion of CUP Decreases the Frequency of Th17 Cells in the Lamina Propria of Large Intestine

**Figure 4:**
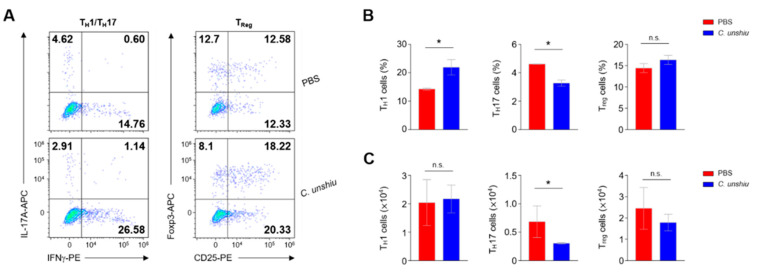
CUP decreases the frequency of T helper 17 (Th17) cells in the lamina propria of large intestine. (**A**) Representative images of the flow cytometry analysis of TH1 (CD45 + CD4 + IL17a-IFNγ+), Th17 (CD45 + CD4 + IL17a + IFNγ−), and Treg (CD45 + CD4 + CD25 + Foxp3+) cells in the lamina propria of large intestine. The summary graphs indicate the frequency (**B**) and absolute numbers (**C**) of immune cells (*n* = 3 per group). All data are represented as the mean ± SEM of independent experiments. Statistical analyses were conducted using unpaired *t*-tests. * *p* < 0.05; n.s., not significant.

To confirm that CUP also relieves the inflammatory response by regulating the population of immune cells, we examined the frequency of diverse subsets of T cells using flow cytometry analysis. First, Th17 and regulatory T (Treg) cells were investigated (Figure 4A). While the administration of CUP decreased the recruitment of Th17 cells to the lamina propria of the large intestine, this treatment did not affect the populations of Treg cells or T helper 1 (TH1) cells (Figure 4B). These findings suggest that CUP inhibits the recruitment of pathogenic Th17 cells.

### 3.5. Bioconversion of CUP Enhances Intestinal Integrity by Upregulating the Levels of Tight Junction Proteins

**Figure 5:**
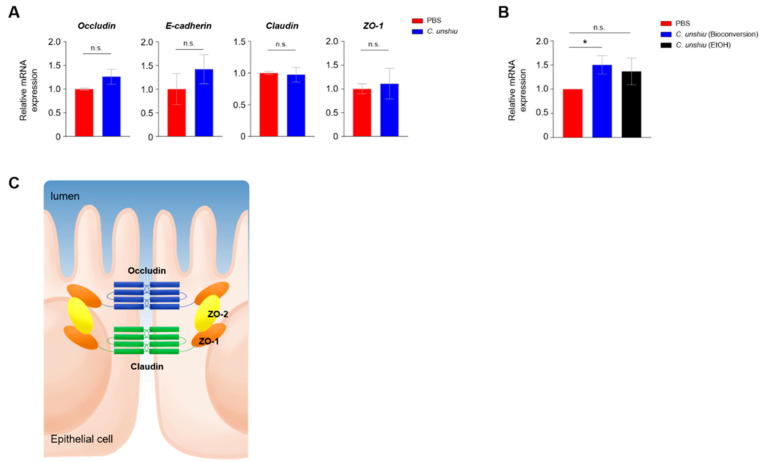
CUP enhances the intestinal integrity. (**A**) Quantitative reverse-transcriptase polymerase chain reaction (qRT-PCR) analysis of the expression levels of tight junction-related genes in Caco-2 cells after treatment with the ethanol extracts of CUP. ZO, zonula occludens. (**B**) The mRNA expression levels of occludin were determined in Caco-2 cells after CUP treatment (bioconversion of CUP). The locations of diverse genes associated with intercellular tight junction are illustrated in panel (**C**). The statistical analyses were conducted using an unpaired one-tailed *t*-test. * *p* < 0.05; n.s., not significant.

To clarify the mechanism of ameliorated inflammation in the CUP-treated mice, we investigated the expression of intracellular tight junctions between IECs, because colitis is closely related to the intensity of intestinal permeability. We first checked the mRNA levels of occludin, E-cadherin, claudins, and ZO-1 in Caco-2 cells 24 h after treatment with ethanol extracts of CUP. No significant differences were observed between the groups; however, the relative expression of occludin and E-cadherin showed an elevated pattern in the CUP-treated Caco-2 cells (Figure 5A). Remarkably, we found that the mRNA expression of occludin was significantly increased only in the bioconversion of CUP but not in the ethanol extracts of CUP (Figure 5B). In summary, these data suggest that CUP biometabolites, but not ethanol extracts, only have protective effects on intestinal permeability. As occludin is located at the apical part of IEC (Figure 5C), we further hypothesized that CUP biometabolites may enhance intestinal epithelial intensity for gut commensal microbiota to prevent infiltration into the lamina propria.

### 3.6. Bioconversion of CUP Alters the Composition of Gut Commensal Bacteria

**Figure 6:**
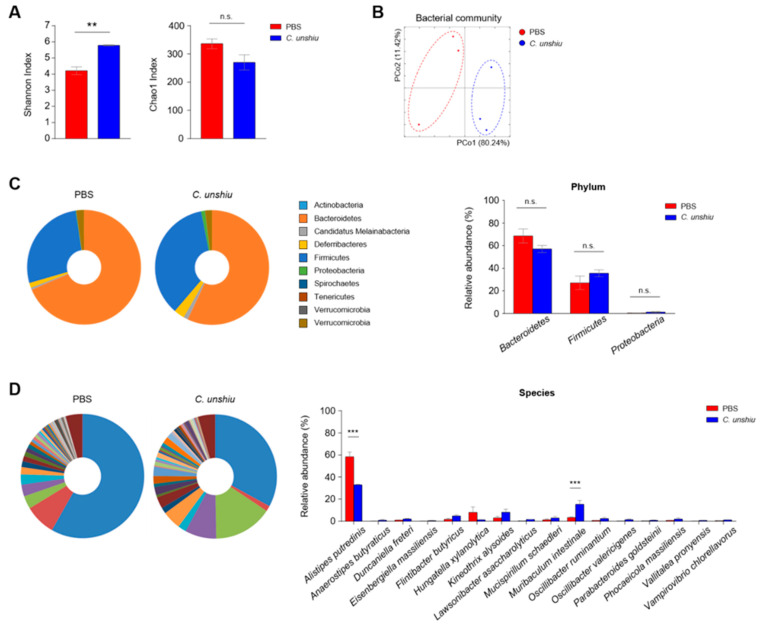
CUP alters the gut commensal microbiota. The community of microbiota in feces of PBS- and CUP-treated mice were analyzed via pyrosequencing. The bacterial composition was analyzed for alpha diversity ((**A**) Shannon and Chao1 index) and beta diversity ((**B**) principal coordinate analysis) between nontreated and treated groups. Representative pie charts (**left**) and relative abundance (**right**) of the commensal bacteria at the phylum (**C**) and species (**D**) levels. All data are represented as the mean ± SEM from three individual mice per group. The statistical analyses were conducted using an unpaired two-tailed *t*-test. ** *p* < 0.01 and *** *p* < 0.001; n.s., not significant.

We first analyzed the fecal microbiome based on the Shannon and Chao1 indices. Interestingly, the Shannon diversity index was higher in the CUP-treated mice than in the control mice, suggesting that the microbial composition of the CUP-fed mice became more even, whereas the Chao1 diversity revealed comparable levels between groups, indicating a similar number of bacterial species regardless of CUP (Figure 6A). In addition, we found a separate community of each fecal microbiome in the principal coordinate analysis (Figure 6B). While the CUP did not differ in composition at the phylum level, including Bacteroidetes, Firmicutes, and Proteobacteria (Figure 6C), we detected reduced Alistipes putredinis and increased Muribaculum intestinale in the CUP-treated mice (Figure 6D). In conclusion, CUP may alter the overall composition of the commensal microbiome without a loss of bacterial quantity.

## 4. Discussion

In this study, we clarified that extraction methods contribute to the different chemical compositions of natural products and the preventive effects of the bioformation of the CUP on severe DSS-induced colitis. The bioconversion of CUP containing hesperetin and naringenin could regulate the NF-κB signaling pathway in Caco-2 cells and diminish pro-inflammatory Th17 cells in murine colon tissue, caused by increased intestinal barrier intensity and altered gut commensal bacteria. Our data suggest that bioconversion metabolites of CUP may affect intestinal homeostasis by regulating epithelial permeability and gut microbiota community.

Flavonoid glycosides, such as hesperidin and narirutin, play pivotal roles in anti-inflammatory and antioxidant activities [48,49] and regulate T-cell populations in various colitis models [50,51,52,53]. Interestingly, our LC-MS analysis revealed that the major components of CUP extracted by the bioconversion method were flavonoid aglycones, such as hesperetin and naringenin, which have been poorly studied for their clinical effects on intestinal diseases. We obtained strong in vivo evidence that the total compounds of the bioconversion metabolites of the CUP, including aglycones, may mitigate DSS-induced intestinal inflammation, which is in line with previous findings that aglycones of natural products regulate host inflammation in the lungs [54], ovaries [55], and liver [56,57].

Different types of CUP influence IκB degradation within canonical NF-κB signaling in Caco-2 cells, although there is still no evidence of an anti-inflammatory effect of the CUP-metabolites on noncanonical NF-κB pathway. The NF-κB induces the expression of pro-inflammatory genes as a transcription factor that triggers an inflammatory response, leading to an impaired intestinal barrier [58,59]. IκB is an inhibitory molecule of NF-κB, implying that the phosphorylation of this molecule brings about the activation of the NF-κB signaling pathway [60]. The inhibition of IκB phosphorylation by blocking IκB kinase (IKK) activity also mitigates the inflammatory response in an IBD model [61]. Although some studies have provided evidence that the inhibition of IKK exacerbates acute inflammation and may impair epithelial integrity [35,62], the ameliorating effects of IKK inhibitors on chronic intestinal inflammation remain unclear. Recent studies have reported that CUP downregulates the expression of pro-inflammatory cytokines by repressing the NF-κB signaling pathway in a macrophage cell line [63,64]. In addition, it is already known that water extracts of CUP decrease phospho-IκB in murine colon tissues [21]. Consistently, our data showed that the bioconversion of the CUP downregulated p50 and phospho-IκB, followed by the recovery of tight junction proteins, whereas the ethanol extracts of the CUP did not mediate this mechanism.

Although a number of CD4+ T cells in the intestinal lamina propria participate in host protection, it is likely that they also contribute to the pathogenesis of IBD. These two-sided effects of CD4 + T cells depend on the cytokines they secrete and how they are stimulated [65]. The supplementation of IL-17a to a *Citrobacter rodentium* infection mouse model relieved the inflammatory response, but the single treatment of IL-33 in the same model showed detrimental effects on the large intestine by preventing the induction of protective Th17 cells [66]. In contrast, inhibiting the infiltration and differentiation of T_H_1 and Th17 cells alleviates the development of inflammation in the colitis model, indicating a pathogenic role of Th17 cells in the intestinal lamina propria [67,68]. Th17 cell-driven proteins also aggravate intestinal fibrosis in a colitis model, indicating a more severe stage of inflammation [69]. Given the pathogenicity of Th17 cells in colitis, it is remarkable that several natural herbs can effectively diminish the frequency of Th17 cells in the intestine [42,70]. Our data also show that CUP is sufficient to decrease Th17 cells in the intestinal lamina propria but not T_H_1 and regulatory T cells.

Our 16S rRNA pyrosequencing data revealed that the administration of CUP metabolites changes the bacterial evenness and composition of the gut commensal community and is associated with significant changes in the abundance of two specific commensal bacteria, *Alistipes putredinis* and *Muribaculum intestinale*. Recent studies have revealed that various flavonoids can modulate gut microbiota and improve microbial dysbiosis in colitis [71] indicating that the gut microbiota plays a crucial role in the pathogenesis of IBD by altering its diversity and abundance [72,73]. In IBD patients, the pathogenesis of IBD has a positive correlation with a decreased abundance of *A. putrednis* and *A. finegoldii* [74,75]. Therefore, a decrease in *A. putredinis* and an expansion of *M. intestinale* in the feces of the CUP-pretreated mice, which showed protective roles against DSS-induced colitis, are consistent with previous studies [75,76,77], suggesting that CUP administration modulates gut microbial composition in a protective way against gut inflammation.

In the past, natural products were not used as medicines due to the fact of their unknown compounds and complicated working mechanisms. Here, we demonstrated that the bioconversion of CUP containing hesperetin and naringenin exerted more powerful effects than ethanol extracts to inhibit NF-κB activation mediated by the gut commensal-intestinal epithelium–Th17 cell axis. Therefore, natural products can be used as prospective therapeutic agents for IBD treatment.

## Data Availability

Not applicable.

## Supplementary Materials

The following supporting information can be downloaded at: https://www.mdpi.com/article/10.3390/nu15020319/s1, Table S1: List of commensal bacteria (species); Table S2: List of specific primer sets used for the real-time PCR in this study.
