# Supplementary material for: Biometabolites of Citrus unshiu Peel Enhance Intestinal Permeability and Alter Gut Commensal Bacteria
Source: Nutrients. 2023 Jan 9;15(2):319. doi: 10.3390/nu15020319 (PMC9862503; doi:10.3390/nu15020319)
Supplement: Supplementary file 1 [file nutrients-15-00319-s001.zip › nutrients-2127443-supplementary.pdf]

Supplementary Materials

Biometabolites of *Citrus unshiu* Peel Enhance Intestinal Permeability and Alter Gut Commensal Bacteria

Se-Hui Lee [1], Dongju Seo [1], Kang-Hee Lee [1], So-Jung Park [1], Sun Park [1], Hyeyun Kim [1], Taekyung Kim [2], In Hwan Joo [3], Jong-Min Park [3], Yun-Hwan Kang [4], Gah-Hyun Lim [1], Dong Hee Kim [3] and Jin-Young Yang [1,*]

[1] Mucosal Immunology Laboratory, Department of Integrated Biological Science, Pusan National University, Busan 46241, Republic of Korea; two_hui@pusan.ac.kr (S.-H.L.); csa83201@pusan.ac.kr (D.S.); rkdgml9056@pusan.ac.kr (K.-H.L.); ahejr26@naver.com (S.-J.P.); sun980610@pusan.ac.kr (S.P.); kelly5060@pusan.ac.kr (H.K.); ghlim16@pusan.ac.kr (G.-H.L.)

[2] Department of Biology Education, Pusan National University, Busan 46241, Republic of Korea;
taekim@pusan.ac.kr

[3] Department of Pathology, College of Korean Medicine, Daejeon University, Daejeon 34520, Republic of Korea; jooih@dju.kr (I.H.J.); jmpark@dju.kr (J.-M.P.); dhkim@dju.kr (D.H.K.)

[4] Department of Industry Promotion, National Institute for Korean Medicine Development, Geongsan 38540, Republic of Korea; k_yunhwan@nikom.or.kr

* Correspondence: jyyang99@pusan.ac.kr; Tel.: +82-51-510-2286; Fax: +82-51-581-2962

Supplementary Table

Table S1. List of commensal bacteria (species). All individual gut microbes shown in the pie chart in Figure 6D are arranged in order of their frequency here.

Alistipes putredinis	*Alistipes onderdonkii*	*Neglecta timonensis*
Hungatella xylanolytica	*Parabacteroides goldsteinii*	*Ruminiclostridium cellulolyticum*
Muribaculum intestinale	*Lawsonibacter asaccharolyticus*	*Anaerosporobacter mobilis*
Kineothrix alysoides	*Oscillibacter valericigenes*	*Anaerostipes butyraticus*
Alistipes senegalensis	*[Clostridium] populeti*	*Anaerotruncus rubiinfantis*
Flintibacter butyricus	*Enterocloster aldenensis*	*Hydrogenoanaerobacterium saccharovorans*
Lacrimispora saccharolytica	*Petrocella atlantisensis*	*Butyricimonas faecihominis*
Mucispirillum schaedleri	*Parabacteroides merdae*	*Roseburia faecis*
Christensenella massiliensis	*Gracilibacter thermotolerans*	*Parasutterella excrementihominis*
Duncaniella freteri	*Vallitalea pronyensis*	*Acetivibrio straminisolvens*
Lacrimispora aerotolerans	*Parabacteroides distasonis*	*[Bacteroides] pectinophilus*
Phocaeicola massiliensis	*Anaeroplasma abactoclasticum*	*Alkalibacter saccharofermentans*
Oscillibacter ruminantium	*Phocea massiliensis*	*Enterocloster asparagiformis*
Christensenella hongkongensis	*Anaerotignum propionicum*	*Lactobacillus johnsonii*
Ligilactobacillus apodemi	*[Clostridium] viride*	*Lachnoclostridium pacaense*
Clostridium cellulovorans	*[Clostridium] polysaccharolyticum*	*Desulfitobacterium hafniense*
Acetivibrio thermocellus	*Eisenbergiella massiliensis*	*[Eubacterium] siraeum*
Vampirovibrio chlorellavorus	*Bacteroides nordii*	*Caecibacterium sporoformans*
Acetatifactor muris	*Desulfovibrio desulfuricans*	*Eubacterium xylanophilum*
Butyricimonas phoceensis	*Mageeibacillus indolicus*	ETC (<0.01%)
Intestinimonas butyriciproducens	*Anaerotaenia torta*	

Table S2. List of specific primer sets used for Real-Time PCR in this study.

Target Gene	Forward Sequence	Reverse Sequence
GAPDH	CAATGACCCCTTCATTGACC	GACAAGCTTCCCGTTCTCAG
Occludin	CCTCCAATGGCAAAGTGAAT	CTCCCCACCTGTCGTGTAGT
E-cadherin	CACCTGGAGAGAGGCCATGT	TGGGAAACATGAGCAGCTCT
Claudin-2	TATGTTGGTGCCAGCATTGT	TCATGCCCACCACAGAGATA
ZO-1	CCACCTCTGTCCAGCTCTTC	CACCGGAGTGATGGTTTTCT
